# A Satellite Explosion in the Genome of Holocentric Nematodes

**DOI:** 10.1371/journal.pone.0062221

**Published:** 2013-04-24

**Authors:** Juan A. Subirana, Xavier Messeguer

**Affiliations:** 1 Departament d’Enginyeria Química, Universitat Politècnica de Catalunya, Barcelona, Spain; 2 Departament de Llenguatges i Sistemes Informàtics, Universitat Politècnica de Catalunya, Barcelona, Spain; University of Oxford, United Kingdom

## Abstract

Centromere sequences in the genome are associated with the formation of kinetochores, where spindle microtubules grow in mitosis. Centromere sequences usually have long tandem repeats (satellites). In holocentric nematodes it is not clear how kinetochores are formed during mitosis; they are distributed throughout the chromosomes. For this reason it appeared of interest to study the satellites in nematodes in order to determine if they offer any clue on how kinetochores are assembled in these species. We have studied the satellites in the genome of six nematode species. We found that the presence of satellites depends on whether the nematode chromosomes are holocentric or monocentric. It turns out that holocentric nematodes are unique because they have a large number of satellites scattered throughout their genome. Their number, length and composition are different in each species: they apparently have very little evolutionary conservation. In contrast, no scattered satellites are found in the monocentric nematode *Trichinella spiralis*. It appears that the absence/presence of scattered satellites in the genome distinguishes monocentric from holocentric nematodes. We conclude that the presence of satellites is related to the holocentric nature of the chromosomes of most nematodes. Satellites may stabilize a higher order structure of chromatin and facilitate the formation of kinetochores. We also present a new program, SATFIND, which is suited to find satellite sequences.

## Introduction

**Figure 1:**
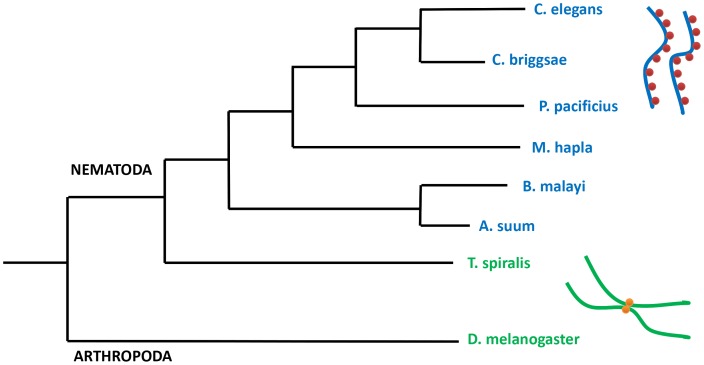
Evolutionary position of the different subphyla of nematodes (adapted from reference 10). Monocentric species have a single large centromere in each chromosome. Holocentric species have centromeres spread over the whole chromosome. Centromeres nucleate the formation of kinetochores, the attaching point of microtubules during mitosis. The holocentric/monocentric nature of the chromosomes is indicated in different colors at the right side of the figure; kinetochores are indicated as red dots.

In this paper we analyze the main features of the satellites found in the genome of nematodes, which are a prototype of holocentric chromosome behavior. We have chosen to study several species for which a substantial amount of the genome has been sequenced. Their phylogenetic relationship is briefly summarized in Figure 1. We should also note that holocentric chromosomes have appeared independently in many species throughout evolution [1] but few genome sequences are available.

Satellite DNA is known to allow the formation of compact heterochromatin typical of centromeres [2]. Examples are found in man [3], [4] and plants [5]. Satellites may give rigidity to the centromeres and facilitate the movement of chromosomes during mitosis [6]–[8]. The structural basis of this effect should be attributed to the repetitive DNA sequence, which will result in a regular position of nucleosomes. Since in monocentric species satellites are usually found in the centromere regions, we may wonder which will be the situation in holocentric nematodes, in which the centromeric function spreads out over the whole chromosomes. The possibility that some satellites play a role in the holocentric behavior of chromosomes has already been suggested by Maddox et al. [8]. However no detailed study on the nature of the satellites found in nematodes is available. In this connection we should note that the centromere specific protein CENP-A has been mapped throughout the genome of *Caenorhabditis elegans* [9]. Quantification reveals that CENP-A is incorporated at low density in domains that cumulatively encompass half the genome. However it is not clear which of the CENP-A rich domains might have a centromeric function. No overall correlation with repeat density was detected.

We should also note that nematodes have a unique chromosome dynamics, with large changes in chromosome number. Frequent losses and acquisitions of new genes are common, including horizontal gene transfers [10]. In this dynamic landscape it appears of interest to determine the distribution of satellites, in particular since in a preliminary study [11] we found evidence suggesting that the reference nematode, *C. elegans*, has a very large number of satellites.

Our study indeed shows that all holocentric nematodes have scattered satellites throughout their genomes. However each nematode species has followed different strategies in building its noncoding genome. No apparent phylogenetic relationships have been found in the repetitive DNA of different holocentric nematodes. On the other hand the monocentric nematode *Trichinella spiralis* stands out by having very few satellites when compared to the holocentric nematode species. For comparison we have also included in our study the euchromatin genome of *Drosophila melanogaster* as an external reference. It belongs to another related phylum (Arthropoda).

Here we will define a satellite as tandem repeats of relatively long sequences, usually in the range 12–200 bases, repeated at least ten times. It should be noted that most of the genomes included in the present study have not been fully sequenced and assembled. In particular repetitive regions are difficult to sequence and position in genomes. However any new feature in the sequenced part of the genomes is a clear positive result. Some clear patterns emerge from our study. On the other hand the absence of some features in any partially sequenced genome has to be interpreted with caution.

## Results

### Overall Features of Satellites in Holocentric Nematodes

The genomes of all the holocentric nematodes we have studied contain a large number of satellites, as shown in Table 1. A complete list is available at our website (http://macrom.upc.edu ). Satellites are related to frequent short motifs, which we have described elsewhere [11]. Here we report our findings for other nematode species. We have found that all holocentric nematode species contain frequent short motifs, with the exception of *T. spiralis.* However their sequence is not conserved, each species has its own motifs, a list is presented in Table 2. A comparison of all motifs is given in Supplementary Table 1. These frequent short motifs may either appear isolated throughout the genome or form long tandem repeats (satellites). Such satellites usually involve additional bases in the repeated motif. Isolated motifs often correspond to the most conserved sequence of large individual repeated motifs, in particular transposons. For example Mo22 is part of Cb000047, an abundant repeat element in *Caenorhabditis briggsae* [12]. This element occurs over twenty thousand times in the genome. It is a Mariner 7 transposon.

**Table 1:** Microsatellites and long satellites.

	Length studied		Total genome	Number of chromosomes		
Species	Size (Mb)	CG %	Approximatesize (Mb)	(contigs)	Long satellites	Satellites/Mb
*C.elegans*	100.3	35.4	100.3	6 (6)	1923	19.2
*C.briggsae*	88.8	37.5	105	6 (6)	2036	22.9
*P.pacificus*	145.9	42.7	169	6 (5106)	1054	7.2
*M.hapla*	53.0	27.4	54	16 (3452)	835	15.8
*B.malayi*	89.2	30.5	93	5 (29808)	4198	47.1
*Brugia>2000*	65.2	30.5	93	5(4931)	518	7.9
*T.spiralis*	58.5	33.9	64	3 (6863)	42	0.7
*D.melanogaster*	120.4	42.4	177	4/5 (6)	171	1.4

The number of long satellites has been determined as described in the main text. It gives those satellites with a maximum repeat size of 200 bases, repeated at least ten times. The data for Brugia>2000 correspond to all the contigs longer than 2000 bases, as explained in the text.

**Table 2:** Frequent short motifs in different nematode genomes.

Motif sequence	Frequency
***C. elegans***	
Motif 10 (Mo10): (TTAGGC)_2_	3418 **S+I**
Motif 11 (Mo11): (ATTTGCCG)_2_	2211 **S+I**
Motif 12 (Mo12): GAAATTCAAATTTT	2821 **S+I**
Motif 13 (Mo13): (ACTACAA)_2_	3169 **S+I**
Motif 14 (Mo14): CAGAGAGTAAAA	2304 **S**
Motif 15 (Mo15): AGCTATATCGTATC	1392 **S**
Motif 16 (Mo16): TTTTCAAAAAAAAA	3782 **I**
Motif 17 (Mo17): TGGCANNNTGCCA	1056 **I**
***C. briggsae***	
Motif 20 (Mo20): (AATTTCWG)_2_	6585 **S+I**
Motif 21 (Mo21): (AATCTCAG)_2_	3505 **S+I**
Motif 22 (Mo22): GTCAACTGATAA	6983 **I**
Motif 23 (Mo23): AAAGATATCAAA	5699 **I**
Motif 24 (Mo24): GCTCAATTATCT	6016 **I**
***P. pacificus***	
Motif 30 (Mo30): (AAAGATC)_2_	7873 **S+I**
Motif 31 (Mo31): GAAATGAAGAGACG	3348 **I**
Motif 32 (Mo32): GAGTTCGAAATACG	2159 **I**
Motif 33 (Mo33): AAAGTGGGCGGAGC	1910 **I**
Motif 34 (Mo34): GAACATTCTAGAAG	1449 **S**
Motif 35 (Mo35): TCTGACCGGTGAGA	1482 **I**
Motif 36 (Mo36): GCGAGAGAGTGTGT	762 **I**
***M. hapla***	
Motif 40 (Mo40): GAAGCATGCTTC	117 **S**
Motif 41 (Mo41): TTTTCCGGAAAG	927 **S**
Motif 42 (Mo42): AAAATCAGAATT	2819 **S+I**
Motif 43 (Mo43): AAATAAACTTGA	1308 **S+I**
Motif 44 (Mo44): AAAATACAAAAA	3238 **S+I**
***B. malayi***	
Motif 50 (Mo50): ACAATATCACTAG 24492 **S**	
Brugia>2000 ACAATATCACTAG	1445 **S**

**S** indicates that the motif takes part in the formation of satellites, usually in.

combination with other sequences.

**I** indicates that the motif is found in isolated positions.

The number of occurrences of these motifs in the other nematode species and in *D. melanogaster* is presented in Table S1.

No frequent short motifs have been found in either *T. spiralis* or *D. melanogaster*.

In *M.hapla* inspection of the most frequent tridecamer motifs shows that none of them contains any C, G bases. The most frequent motifs are AAAAAAATTTTTT (10056 cases) and AAAAATATTTTTT (10074 cases). The latter motifs do not form any significant cluster, they are scattered throughout the genome. In this table we only include those frequent short motifs in *M. hapla* which contain some C, G bases.

**Figure 2:**
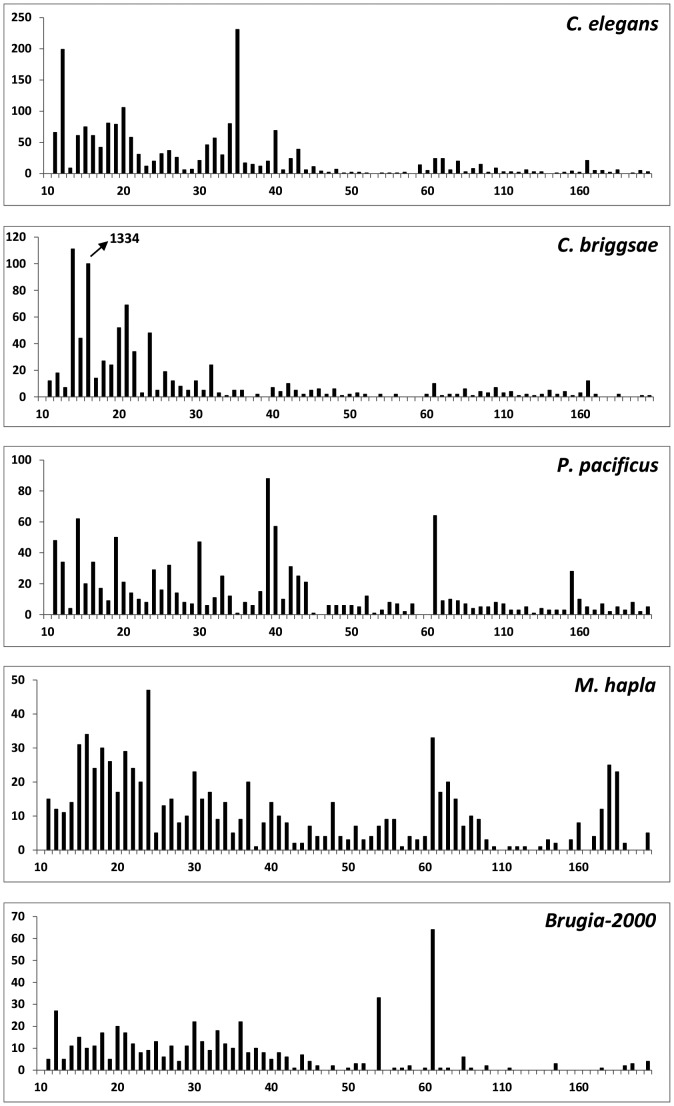
Distribution of motif sizes in the satellites of different nematodes. For sequences longer than 60 bases the data have been merged in bins of 5 bases. The percentage of short motifs (<50 bases) is the largest in all cases, with up to 95% in *C.briggsae*. Hexadecamer motifs are extremely abundant in this species; their actual frequency is indicated by an arrow in the figure.

In the following sections we will briefly review the main features of satellites in each species, which we summarize here. The distribution of motif size (Figure 2), sequence and total length of satellites varies in each species. It is striking that in spite of the general occurrence of scattered satellites in all these genomes, very little conservation of properties is found. In particular the base sequence of the repeated motifs found in each species varies, as shown in Table 2 and in Table S1. We should note that very few satellites correspond to coding sequences, such as repeated amino acid sequences in proteins.

In all species long satellites (>2 Kb) are less frequent. For example, in the complete genome of *C. elegans* we find that 338 satellites have a length between 2 and 5 Kb and 70 are longer than 5 Kb. These values represent 17.6% and 3.6% of the total number of satellites. The relative frequency of long satellites in the other nematode species is even lower.

The size of repeated motifs presents a unique distribution in each species, but some general trends are apparent. Satellites with short repeated motifs (<50 bases) predominate, within the limits of 69% for *M. hapla* and 95% for *C. briggsae*. In Figure 2 we present a comparison of the frequency of satellite motifs, which clearly varies in each species. In all cases the distribution of motif sizes is not smooth, but contains clear peaks, which correspond to related sequences. We should note that the relative frequency of satellite motifs presented in Figure 2 is not expected to vary significantly when whole genome frequencies become available.

### Satellites in the Rhabditina

We have studied three species in this clade. The results obtained with *C. elegans* are more accurate, because its genome has been fully sequenced. Their total length is approximately 2.5 Mb; their average composition is the same as the total genome. Some of these satellites belong to the rapidly renatured DNA already detected in the pioneering study of Sulston and Brenner [13]. The repeated sequences in this organism can be visualized by browsing through the genome, as presented in Wormbase (http://www. wormbase.org). Satellites are not evenly distributed throughout the genome, they accumulate at both ends of each chromosome.

As found in all nematodes, the most common motif sizes are smaller than 50 bases. Sizes of 12 and 34–35 bases predominate. The 12 base repeat corresponds to telomere repeats (Mo10) scattered throughout the genome, as already described [11], [14]. On the other hand it is not clear what favors the 34–35 motif length in many satellites, it is present in all chromosomes. Most of the latter motifs have a related sequence.

The genomes of *C. briggsae* and *Pristionchus pacificus* also present frequent satellites and repeated short motifs, as shown in Tables 1 and 2. Both species contain a significant number of long satellites, over 5 Kb. The length of the repeated motifs in satellites is very different in each species. In *C. briggsae* about two thirds of the satellites have a hexadecamer repeat, often formed by two octamers, with a sequence related to Mo20 and Mo21. It is interesting to note that hexadecamer repeats have a much lower frequency in all other nematodes.

In *P. pacificus* the distribution of motif lengths is very heterogeneous: it has 53 satellites with a 65 base repeated motif and 88 satellites with a 39 base repeated motif. In fact Mo34 is only found in such satellites with a 39 base repeated motif. The genome of this species is very incomplete, about 14% remains un-sequenced. Thus the number of satellites detected in this species is only a lower limit (Table 2).

### Satellites in *Meloydogine hapla*

*M. hapla* has several unique features among the nematodes we have studied:. It has a small genome, which has been sequenced over 95% (Table 1). It has not been assembled, but its contigs have no unplaced residues. It has the highest A,T% of all the nematodes we have studied. It also presents abundant losses and gains of genes, when compared with other nematode species [10]. It has very few microsatellites, as reported by Castagnone-Sereno et al. [15], but the number of satellites is similar to other species. However most satellites are rather short, no satellite longer than 5 Kb has been detected. A puzzling feature of this species is the absence of the telomeric repeat Mo10, in spite of the fact that it has a comparatively large number of chromosomes.

### Satellites in *Brugia malayi*

The genome sequence available for *Brugia malayi* must be analyzed with care It contains many short contigs which may have redundant information. About 28.3% of the sequence data are found in contigs shorter than 2 Kb. We have carried out our calculations both with the whole contigs file and with a shorter file (Brugia>2000) which only contains all contigs longer than 2 Kb.

Most satellites in this species are part of a specific family with a mixture of related motifs 62 and 54 bases long. Mo50 is part of these motifs. A typical satellite is given in the supplementary material (Table S2). Among the total number of 4198 satellites, 2768 correspond to satellites with the 54/62 motifs. In the shorter Brugia>2000 file we found 518 satellites, but only 95 of which correspond to satellites with these motifs. Since short contigs probably contain redundant information, the actual value should be intermediate between the two values mentioned. Most of the other satellites (382) have motif lengths in the range 11–46 bases. For comparison we have placed the data for Brugia>2000 in Figure 2.

### Satellites in *Trichinella spiralis*

In the case of *T. spiralis* we only found a small number of satellites, as it is apparent in Table 1. No frequent short motifs were found. The satellites were small, less than 1 Kb, and the repeated motif was in the range 12–32 bases.

This species stands out among all the nematodes studied for the low number of satellites present, it is closer to *D*. *melanogaster* in this respect. It is also the only nematode we have studied which has monocentric chromosomes [16]. In the report describing the genome sequence of this species no mention is made of the eventual presence of either telomeric repeats (Mo10) or centromeric satellites [17]. Most likely they were not sequenced.

### Satellites in *Drosophila melanogaster*

*D. melanogaster* has been taken as an example of a species with monocentric chromosomes. It belongs to the arthropoda, a protostome phylum, close to the nematoda. Its centromeric regions have not been fully sequenced, but it is known that they mainly contain microsatellites [18]. On the other hand its euchromatin genome has been fully sequenced. As reported before [11], it does not contain frequent repeated short motifs throughout its genome. Our search for satellite sequences only yielded a few cases, as shown in Table 1. Most satellites correspond to transposons and repeated amino acid sequences in large proteins, so that their origin in the genome may be unrelated to the satellite sequences found in holocentric nematodes.

## Discussion

### Unique Satellite Features in Holocentric Nematodes

Our results show that isolated short motifs and long satellites are a common unique feature in all holocentric nematodes. Recently a large number of satellites have also been found in a holocentric plant, *Luzula elegans* [19]. It appears that these organisms have developed similar mechanisms in order to maintain satellites distributed throughout their genomes. In the case of holocentric nematodes each species uses different motifs for this purpose. No apparent relation has been found with the centromeric satellite sequences present in other eukaryotes [3]–[5].

Phylogenetic relations among nematodes have been determined by the study of different markers [10], [20], [21]. We expected to find some phylogenetic relations among satellites, but we did not find any clear sequence conservation.

We may wonder about the origin of the abundant satellites in holocentric nematodes. A possible evolutionary scenario is that early nematodes had monocentric chromosomes. Later in evolution, while nematodes were invading new habitats and new hosts, they started to loose and gain many genes [10]. At the same time, the repetitive centromeric DNA sequences broke down and satellite DNAs spread throughout whole chromosomes, which later underwent independent evolution in each nematode species.

### Telomeres

Telomeres have only been sequenced in *C. elegans*. Therefore it is not possible to know the detailed composition of telomeres in other nematode species. However we should note that the Rhabditina, *A. suum* and *B. malayi* contain a significant amount of the standard nematode telomere sequence TTAGGC (Mo10), as shown in Table S1-a. This motif is found as clusters/satellites in all these species. The number of repeats is lower than in *C. elegans*, most likely due to incomplete sequencing. So we may assume that the species mentioned do use the same sequence in their telomeres, which has been described in detail in *A. suum* [22].

On the other hand no clusters of the standard telomere sequence have been found in either *M. hapla* or *T. spiralis*. It remains to be established which is the telomere structure in such species. No alternative repeat with strings of Gs has been detected in their genomes. It is tempting to speculate that telomeres in these species might have a complex telomere structure, as described in many fungi [23].

### Conventional Genomic Features of the Monocentric *T. spiralis*

*T. spiralis* is unique among the nematodes we have studied. It has monocentric chromosomes and does not undergo the massive change of genes characteristic of most nematodes [10]. In this respect this species is considered to be slowly evolving [24], with a genome closer to the primitive ancestor of Arthropoda and Nematoda. The monocentric *T. spiralis* and *D. melanogaster* apparently conserve features of their common ancestor: they have not evolved unique abundant satellites in their euchromatin. It would be of interest to know if there is any species in other nematode clades which has monocentric chromosomes.

### Any Role for Satellites in Holocentric Chromosomes?

The possibility that some satellites play a role in the holocentric behavior of chromosomes should be considered, as already mentioned in the introduction. Satellites may give rigidity to the chromosomes and facilitate their movements during mitosis. The structural basis of this effect should be attributed to the repetitive DNA sequence, which will result in a regular position of nucleosomes. Our results indicate that the sequence and size of the repetitive motifs may not be important for this function. Kinetochores appear at several places in holocentric chromosomes, but the signals that determine their formation are unknown, as recently reviewed by several authors [25], [26]. From the studies of Albertson and Thomson [27] it is possible to estimate that there are approximately twenty kinetochores in each *C. elegans* chromosome. It is not known if kinetochores form either at random or at specific regions of the chromosomes. Nevertheless Gassmann et al. have recently reported [9] that the centromeric protein CENP-A is found in domains with a variable size, which encompass half the genome. Some satellites may contribute to the establishment of kinetochores in such domains.

## Materials and Methods

### Genome Sequences

Most genome sequences were downloaded from GenBank (http://www.ncbi.nlm.nih.gov/genomes/leuks.cgi), with the exceptions of *Ascaris suum* (ftp://ftp.wormbase.org/pub/wormbase/species/a_suum), *M. hapla* (http://www.hapla.org ) and *P. pacificus*. The latter was downloaded as an artificial single chromosome (http://genome.ucsc.edu/). Most genome sequences are incomplete and contain many gaps, in which long string of Ns have been placed and are part of the published sequences. An exception is the genome of *C. elegans*, which is considered to be complete. In the case of *D. melanogaster* only the euchromatin part has been fully sequenced. In the case of *T. spiralis,* the sequence available in GenBank contains the scaffolds. All of them have gaps. However the contigs file has no unplaced bases. It was obtained from the University of Washington (http://genome.wustl.edu/).

Unfortunately the data available for *A. suum* are not suitable for the study of satellites: the published sequence contains about 24000 very short contigs (100–500 bases), which can not contain complete satellites. For this reason we have not included this species in our study.

### Computer programs

Repetitive sequences were analyzed with the programs MREPATT [28] and CONREPP [11]. A new program (SATFIND) was also developed in order to determine the position of long tandem repeats (satellites). It finds the localization of clusters of any short sequence (<12 bases) repeated a minimum number of times in regions with a fixed size. SATFIND can be downloaded from our website (http://alggen.lsi.upc.edu).

In this paper we have used the SATFIND program in order to determine the presence of at least ten repeats of any decamer sequence in regions 2 Kb long. In this way motifs of 10–200 bases repeated at least 10 times can be positioned in any genome. With these limits, short motifs (6–9 bases) repeated at least 20 times are also localized. The program eliminates microsatellites with short repetitive motifs (1–5 bases). The advantage of this method is that it only requires that the repeats have ten identical bases, the rest of the repeat may be highly variable, including point mutations and indels. Those repeats which showed no clear consensus were discarded. Note that this definition of satellite is very different from the original definition derived from density gradient centrifugation [2]. Often the DNA sequence in such satellites was found to be very heterogeneous.

The output of the program includes:

- The sequence of 10 bases which has been found repeated at least 10 times in a region of 2 Kb.
- The number of tandem repeats found.
- The genome coordinates of the region which contains the satellite.
- The length of genome covered by the satellite, which may be longer than the initial 2 Kb, since the program continues searching when repeats are found beyond the end of the 2 Kb. The length detected by the program occasionally is longer than the actual satellite. It happens when the repeated 10 base motif is found embedded in unrelated sequences in the neighborhood of the satellite.
- The most frequent size of the motif repeated in tandem in the satellite.
- In a second output file we give the sequence of the repeated motifs in all satellites. If the repeated motifs show a large variation in size, the satellite is eliminated. In this work we have chosen to accept only those satellites in which 40% of the motifs have a similar size. Thus we eliminate from the output some satellites which are very irregular.

With this method we include in our satellite list some tandem repeats with short motifs (6–9 bases) which should be considered as microsatellites. However they are only a small number among the satellites we have found. In general we have not attempted to identify the few satellites which are found in coding regions. If satellites play a structural role in chromosome behavior, their location in coding regions is not critical.

## Supporting Information

Table S1 **Comparison of the frequency of short repeated motifs in different nematode species. It has three separate sections.**
(DOC)

Table S2 **Example of one of the abundant satellites in** ***B. Malayi*** **.**
(DOC)
