# Supplementary material for: A Satellite Explosion in the Genome of Holocentric Nematodes
Source: PLoS One. 2013 Apr 24;8(4):e62221. doi: 10.1371/journal.pone.0062221 (PMC3634726; doi:10.1371/journal.pone.0062221)
Supplement: Table S1 — Comparison of the frequency of short repeated motifs in different nematode species. It has three separate sections. (DOC) [file pone.0062221.s001.doc]

**Table S1-a. Repeated short motifs in *C.elegans***

| **Species** | **Mo10** | **Mo11** | **Mo12** | **Mo13** | **Mo14** | **Mo15** | **Mo16** | **Mo17** |
| --- | --- | --- | --- | --- | --- | --- | --- | --- |
| *C.elegans* | 3418 | 2211 | 2821 | 3169 | 2304 | 1392 | 3782 | 1098 |
| *C.briggsae* | 112 | 0 | 304 | 560 | 14 | 0 | 2043 | 327 |
| *P.pacificus* | 147 | 0 | 28 | 269 | 21 | 0 | 358 | 567 |
| *B.malayi* | 331 | 0 | 44 | 4 | 30 | 1 | 879 | 36 |
| *A. suum* | 173 | 0 | 89 | 17 | 74 | 0 | 1615 | 636 |
| *D.melanogaster* | 21 | 2 | 43 | 11 | 19 | 0 | 134 | 199 |

The number of motifs found in each genome includes the complementary sequence. All these sequences are practically absent in the other nematodes we have studied (*M. hapla* and *T. spiralis*). The following sequences are shown:

Motif 10 (Mo10): (TTAGGC)_2_

Motif 11 (Mo11): (ATTTGCCG)_2_

Motif 12 (Mo12): GAAATTCAAATTTT

Motif 13 (Mo13): (ACTACAA)_2_

Motif 14 (Mo14): CAGAGAGTAAAA

Motif 15 (Mo15): AGCTATATCGTATC

Motif 16 (Mo16): TTTTCAAAAAAAAA

Motif 17 (Mo17): TGGCANNNTGCCA

**Table S1-b. Repeated short motifs in *C.briggsae* and *P.pacificus***

The number of motifs found in each genome includes the complementary sequence. All these sequences are practically absent in the other nematodes we have studied. The following sequences are shown:

| **Species** | **Mo20** | **Mo21** | **Mo22** | **Mo23** | **Mo24** | **Mo30** | **Mo31** | **Mo32** | **Mo33** | **Mo34** | **Mo35** | **Mo36** |
| --- | --- | --- | --- | --- | --- | --- | --- | --- | --- | --- | --- | --- |
| ***C. elegans*** | 21 | 2 | 96 | 126 | 18 | 32 | 8 | 0 | 248 | 7 | 0 | 21 |
| ***C.briggsae*** | 6585 | 3505 | 6983 | 5699 | 6016 | 5 | 5 | 0 | 1152 | 19 | 0 | 24 |
| ***P.pacificus*** | 2 | 0 | 18 | 82 | 34 | 7873 | 3348 | 2159 | 1910 | 1449 | 1482 | 1886 |
| ***D.melanogaster*** | 2 | 1 | 25 | 79 | 17 | 4 | 0 | 0 | 3 | 0 | 0 | 9 |

Motif 20 (Mo20): (AATTTCWG)_2_

Motif 21 (Mo21): (AATCTCAG)_2_

Motif 22 (Mo22): GTCAACTGATAA

Motif 23 (Mo23): AAAGATATCAAA

Motif 24 (Mo24): GCTCAATTATCT

Motif 30 (Mo30): (AAAGATC)_2_

Motif 31 (Mo31): GAAATGAAGAGACG

Motif 32 (Mo32): GAGTTCGAAATACG

Motif 33 (Mo33): AAAGTGGGCGGAGC

Motif 34 (Mo34): GAACATTCTAGAAG

Motif 35 (Mo35): TCTGACCGGTGAGA

Motif 36 (Mo36): GCGAGAGAGTGTG

**Table S1-c. Repeated short motifs in *M. hapla* and *B.malayi***

| **Species** | **Mo40** | **Mo41** | **Mo42** | **Mo43** | **Mo44** | **Mo50** |
| --- | --- | --- | --- | --- | --- | --- |
| *C.elegans* | 11 | 58 | 400 | 148 | 1363 | 4 |
| *C.briggsae* | 3 | 62 | 577 | 74 | 938 | 1 |
| *P.pacificus* | 8 | 40 | 129 | 71 | 533 | 5 |
| *M.hapla* | 117 | 927 | 2819 | 1308 | 3238 | 1 |
| *B.malayi* | 2 | 47 | 111 | 117 | 700 | 24492 |
| *Brugia-2000* | nd | nd | nd | nd | nd | 1445 |
| *A. suum* | 32 | 149 | 157 | 411 | 795 | 11 |
| *T.spiralis* | 4 | 9 | 80 | 63 | 435 | 0 |
| *D.melanogaster* | 3 | 13 | 85 | 114 | 762 | 1 |

The number of motifs found in each genome includes the complementary sequence. The motifs in *M.hapla* are ordered by their increasing content in A,T.

Motif 40 (Mo40): GAAGCATGCTTC

Motif 41 (Mo41): TTTTCCGGAAAG

Motif 42 (Mo42): AAAATCAGAATT

Motif 43 (Mo43): AAATAAACTTGA

Motif 44 (Mo44): AAAATACAAAAA

Motif 50 (Mo50): ACAATATCACTAG
