# Supplementary material for: A Satellite Explosion in the Genome of Holocentric Nematodes
Source: PLoS One. 2013 Apr 24;8(4):e62221. doi: 10.1371/journal.pone.0062221 (PMC3634726; doi:10.1371/journal.pone.0062221)
Supplement: Table S2 — Example of one of the abundant satellites in B. Malayi . (DOC) [file pone.0062221.s002.doc]

**Table S2**

**Example of one of the abundant tandem repeats in Brugia Malayi, Contig 2186**

**84 repeats, about half with 62 bases, the rest shorter, mainly with 54 bases. The alignment covers the whole contig, which has 4911 bases.**

>gi|154233630|gb|AAQA01002186.1| Brugia malayi ctg_55001, whole genome shotgun sequence

ATATAACAAGATCA

CTAGAAAATATTTTGATTAATTCATTCCCCTTCTCTGCATATATAACAATATCA

CTAGAAGACATATTGATTAATTCATTCCCCTTCTCTGCATATATAACAATATCA

CTAGAAGACATTTTGATTAATTCATTAACTCRCACCATATCACTACAGATATAACAATATCA

CTAGAAGATATTTTGATTAATTCATTAACTCATACCATTTCTCTGCAGATATAACAATATCA

CTACAAGACGTTTTGATCACTTCATTAACTCATACCATATCTCTACAGATATAACACTATCA

CTAGAAGACATTTTGATTAATTCATTCCCCTTCTCTACAGATATAACAATATCA

CTAGAAGACATTTTGATTGCTTCATTAGCTCATACCATTTCTCTACAGATATAACATTATCA

CTAGAAGACATATTGATTAATTCATTCACCTTCTCTACAGATATAACAATATCA

CTAGAAGACATATTGATTAATTCATTCCCCTTCTCTACAGATTTAACAATATCA

CTAGAAGACGTTTTGATTACTTCATTAACTCACACCATATCACTACAGATATAACAATATCA

CTAGAAGATATTTTGATTAATTCATTAACTCATACCATTTCTCTGCAGATATAACAATATCA

CTACAAGACGTTTTGATCACTTCATTAACTCATACCATATCTCTACAGATATAACACTATCA

CTAGAAGACATTTTGATTAATTCATTCCCCTTCTCTACAGATATAACAATATCA

CTAGAAGACATTTTGATTGCTTCATTAGCTCATACCATTTCTCTACAGATATAACATTATCA

CTAGAAGACATATTGATTAATTCATTCACCTTCTCTACAGATATAACAATATCA

CTAGAAGGCATATTGATTAATTCATTCCCCTTCTCTACAGATATTACAAAATCA

CTAGAAGACGTTTTGACTGCTTCATTAAGTCATGCCATATCTCTACAGATATAACATTATCA

CTAGAAGACATTTTGATTAATTCATTCCCCTTCTCTACAGATATAACAATATCA

CTAGAAGACGATTTGATTACTTCATTAACTCATTCCATTTCTCTGCAGATATAACAATATCA

CTAGAAGACATTTTGATTAATTCATTAGCTCATACCATTTCTCTACCAATACAACAAGATCA

CTAGAAGATATTTTGATTACGTCATTAACTCATACCATTTCTCTACAGATATAACAATATCA

CTAGAAGACGTTTTGATTACTTCATTAACTCATACCATATCTCTACAGATATAACAAGATCA

CTAGAAGACATTTTGATTAATTCATTCCCCTTCTCTGCATATATAACAATATCA

CTAGAAGACGTTTTGATTACTTCATTAACTCATACCATATCTCTACAGATATAACAAGATCA

CTAGAAGACATATTGATTAATTCATTCACCTTCTCTACAGATATAACAAKATCA

CTAGAAGACATWTTGATTAATTCATTCCCCTTCTCTRCAKATWTAACAATATCA

CTAGAAGACGTTTTGATTACTTCATTAACTCAYACCATATCWCTACAGATATAACAAKATCA

CTAGAAGAYATWTTGATTAATTCATTAACTCATACCATTTCTCTACAGATATAACAATATCA

CTAGAAGACATTTTGATAACTTCATTAACTCATACCATATCTCTACCAGTATAACAAGATCT

CAAGAAGATATTTTGATTACTTCATTAACCAATACCATTTCACTACAGATATAACAAGATCA

CTAGAAAATATTTTGATTAATTCATTCCCCTTCTCTGCATATATAACAATATCA

CTAGAAGACATTTTGATTAATTCATTAACTCACACCATATCACTACAGATATAACAATATCA

CTAGAAGATATTTTGATTAATTCATTCCCCTTCTCTACAGATATAACAATATCA

CTAGAAGACATTTTGATTGCTTCATTAGCTCATACCATTTCTCTACAGATATAACATTATCA

CTAGAAGACATTTTGATTAATTCATTCCCCTTCTCTACAGATATAACAATATCA

CTAGAAGGCATATTGATTAATTCATTCCCCTTCTCTACCAGAGATATTACAAAATCA

CTAGAAGACGTTTTGACTGCTTCATTAAGTCATGCCATATCTCTACAGATATAACATTATCA

CTAGAAGACATTTTGATTAATTCATTCCCCTTCTCTACAGATATAACAATATCA

CTAGAAGACGTTTTGATTACTTCATTAACTCATACCATATCTCTACAGATATAACAAGATCA

CTAGAAGACATTTTGATTAATTCATTCCCCTTCTCTGCATATATAACAATATCA

CTAGAAGACGTTTTGATTACTTCATTAACTCATACCATATCTCTACAGATATAACAAGATCA

CTAGAAGACATATTGATTAATTCATTCCCCTTCTCTACAGATATAACAATATCA

CTAGAAGACATTTTGATTAATTCATTCCCCTTCTCTCCAGATATAACAATGTCA

CTAGAAGACGTTTTGATAACTTCATTAACTCATACCATATCTCTACCAGTATAACAAGATCT

CAAGAAGATATTTTGATTACTTCATTAACCAATACCATTTCACTACAGATATAACAAGATCA

CTAGAAAATATTTTGATTAATTCATTCCCCTTCTCTGCATATATAACAATATCA

CTAGAAGACATATTGATTAATTCATTCCCCCTTCTCTGCAKATWTAACAATATCA

CTAGAAGACRTTTTGATTAMTTCATTAACTCACACCATATCACTACAGATATAACAATATCA

CTAGAAGATATTTTGATTAATTCATTAACTCATACCATTTCTCTGCAGATATAACAATATCA

CTACAAGACGTTTTGATCACTTCATTAACTCATACCATATCTCTACAGATATAACACTATCA

CTAGAAGACATTTTGATTAATTCATTCCCCTTCTCTACAGATTTAACAATATCA

CTAGAAGACGTTTTGATTACTTCATTAACTCACACCATATCACTACAGATATAACAATATCA

CTAGAAGGCATTTTGATTATTTCATTAACTCATACCATTTCTCTGCAAATATAACAATATCA

CTAGAAGACATTTCGATCAATTCATGCCACTTCTCTACTGATAAAACAATATCA

CTAGAAGACATTTTGATTTAATCATTAACTCATACCATTTCTCTACAGATTTAACAATATCA

CTAGAAGACATTTTGATTAATTCACGCCATTTCTCTGCAAATATAACAATATCA

CTAGAAGACATTTCGATCAATTCATGCCACTTCGCTGCTGATATAACAATATCA

CTAGAAGACATTTTGATTAATTCATTAACTCCTACCATTTCTCTGTTGATACAACAACATCA

CTAGAAGACATTTTGATTAATTCATTAACTCATACCATTTCTCTGTTGATACAACAACATCA

CTAGAAGACATTATGATTAATTCATTAACTCATACCATTTCTCTGCAAACATAACAATATCA

CTAGAAGAGATTTTGATTCTCTACAGATTAAACAATATCG

ATAGAAGACATTTTTTTTTTTTATTTCATGTCACTTCGCTGCTGATATAACAATATCA

CTAGAAGGCATTTTGATTATTTCACTAACTCATACCATTTATTAATCAATATAACAAGATCA

ATTGGAGACATTTTGATTTATTCATTTACTCATACCATTTCTCTACAGATTAAACAATATCA

CTAGAAGACATTTTGATTAAATCATGAACTCATACCATTTCTCTACAGATTTAACAATATCA

CTAGAAGACATTTTGATTAATTCACGCCATTTCTCTGCAAATATAACAATATCA

CTAGAAGACATTTCGATCAATTCATGCCACTTCGCTGCTGATATAACAATATCA

CTAGAAGGCATTTTGATTATTTCATTAACTCATACCATTTCTCTGCAAATATAACAATATCA

CTAGAAGACATTTTGATTAATTCATTAACTCATACCATTTCTCTGCAAATATAACAATATCA

CTAGAAGACATTTTGATTAATTCATTAACTCCTACCATTTCTCTGTTGATACAACAACATCA

CTAGAAGACATTTTGATTAATTCATTAACTCATACCATTTCTCTGCAAACATAACAATATCA

CTAGTAGAGATTTTGATTCTCTACAGATTAAACAATATCA

CTAGAAGACATTTTTTTTTTTATTTCATGTCACTTCGCTGCTGATATAACAATATCA

CTAGAAGGCATTTTGATTATTTCACTAACTCATACCATTTATTAACCAATATAACAAGATCA

ATTGGAGACATTTTGATTTATTCATTTACTCATACCATTTCTCTACAGATTAAACAATATCA

CTGGAAGACATTTTGATTAAATCATAAACTCATACCATTTCTCTACAGATTTAACAATATCA

CTAGAAGACATTTTGATTAATTCACGCCATTTCTCTGCAAATATAACAATATCA

CTAGAAGACATTTCGATCAATTCATGCCACTTCTCTACTGATAAAACAATATCA

CTAGAAGGCATTTTGATCAATTCATGCCACTTCTCTACTGATAAAACAATATCA

CTAGAAGACATTTTGATTAAATCATTAACTCATACCATTTCTCTACAGATTAAACAATATCA

ATAGAAGACATTTTGATTAATTCACGCCATTTCTCTGCCAATATAACAATATCA

CTAGAAGACGTTTTGATTAATTCATTAACTCATACCATTTCTCTGCAAATATAACAATATCA

CTAGAAGACATTTTGATTAATTCATTTACTCATACCATTTCTCTGCAAACATAACAATATCA

AAAGAAGACATTTTTTTTTTTATTTCATGTCACTTCGCTGGTGATATAACAATATC
